# 12-month SARS-CoV-2 antibody persistency in a Tyrolean COVID-19 cohort

**DOI:** 10.1007/s00508-021-01985-x

**Published:** 2021-11-23

**Authors:** Florian Deisenhammer, Angelika Bauer, Chiara Kavelar, Dagmar Rudzki, Annika Rössler, Janine Kimpel, Wegene Borena, Markus Reindl

**Affiliations:** 1 Neuroimmunology Laboratory, Innrain 66, 2nd floor, 6020 Innsbruck, Austria; 2 Department of Neurology, Medical University of Innsbruck, Innsbruck, Austria; 3 Institute of Virology, Department of Hygiene, Microbiology and Public Health, Medical University of Innsbruck, Innsbruck, Austria

**Keywords:** Immunity, Prospective, ELISA, Virus, Neutralizing

## Abstract

**Background:**

Short-term antibody response to severe acute respiratory syndrome coronavirus 2 (SARS-CoV-2) has been shown previously. The further development remains to be determined.

**Methods:**

We prospectively followed 29 coronavirus disease 2019 cases, mean age 44 ± 13.2 years. Except for one participant in whom rheumatoid arthritis existed, all other cases were previously healthy. We determined anti-viral binding antibodies at 2–10 weeks, 3 months, 6 months, and 12 months after disease onset as well as neutralizing antibodies (NAb) against wild type at 6 and 12 months and the B.1.1.7 and B.1.351 variants at month 12. Three binding antibody assays were used, targeting the nucleocapsid protein (NCP), the S1 subunit of the spike protein, and the receptor binding domain (RBD).

**Results:**

Antibodies to the RBD persisted for 12 months in all cases with increasing concentrations, whereas antibodies to S1 dropped below cut-off point in 7 participants and NCP antibodies were above cut-off point in only 5 subjects at month 12. The NAb against wild type were detected in all but 2 samples at 12 months of follow-up but clearly less frequently when targeting the variants. In 5 participants who were vaccinated against COVID-19 there was a strong increase of antibodies against S1 and RBD as well as an increase of NAb titres against wild type and the variants.

**Conclusion:**

There was a persisting antibody response against SARS-CoV‑2 up to 12 months after COVID-19 with declining concentrations except for RBD and a strong increase of all antibody concentrations after vaccination.

**Supplementary Information:**

The online version of this article (10.1007/s00508-021-01985-x) contains supplementary material, which is available to authorized users.

## Introduction

Coronavirus disease 2019 (COVID-19) caused by severe acute respiratory syndrome coronavirus 2 (SARS-CoV-2) leads to an acute immune response transforming into immune memory protecting from infection in previously infected or vaccinated persons [1]. Immunity as a clinical outcome is hard to determine in open-label epidemiological settings. Therefore, surrogates are commonly used mostly by measurement of agent-specific antibodies as a component of immune memory [2]. The short-term antibody response has been shown in COVID-19 cases in several reports [3, 4]; [5–9], and in few long-term observations up to 12 months [10]. Here we present the 12-month follow-up SARS-CoV‑2 antibody results in a longitudinal prospective Tyrolean cohort which was the primary goal of the study. Additionally, we present pre-vaccine versus post-vaccine SARS-CoV‑2 antibody responses in a subset of 5 study subjects.

## Material and methods

### Study population

The cohort comprised 29 participants (14 females and 15 males) as previously described [3] with an average age of 44±13.2 years. All but one asymptomatic case had symptomatic COVID-19 with mild to moderate disease course and full recovery except for one person with persistent dysosmia.

All cases occurred in March and April 2020 and had a positive SARS-CoV‑2 antibody test in April 2020 at the latest. As defined by the original prospective study protocol, blood samples were serially collected at 4 time points after symptom onset, T1 between 2 weeks up to 2 months, T2 between 3 and 4 months, T3 at 6 months, and T4 at 12 months. Binding SARS-CoV‑2 antibodies were determined at all time points and neutralizing antibodies against the wild type (Wuhan) were done in all samples at T3 and T4. Additionally, neutralizing antibodies against the variants B.1.1.7 (alpha) and B.1.351 (beta) were tested at T4.

In three of five study participants who received COVID-19 vaccines coincidentally (and therefore constitutes a post hoc secondary endpoint) shortly before the last follow-up, samples were drawn immediately before vaccination and in 2 participants T3 was the last follow-up before vaccination. Therefore, the latter two persons were excluded from the T4 analysis for antibody persistency after infection. We also analyzed the change of antibody response in the five participants before and after vaccination including neutralization of the variants B.1.1.7 (alpha) and B.1.351 (beta).

### Assays

In addition to T4 samples we reanalyzed all samples collected at T1, T2, and T3 from a previous study [3] for binding antibodies, as assays were modified, improved, and added. All binding assays are CE certified, i.e. fully validated.

### S1 subunit of SARS-CoV-2 spike protein ELISA

Serum IgG antibodies were determined by a commercial ELISA (Euroimmun, Lübeck, Germany, Catalogue # EI 2606-9601–10 G), which includes the new WHO reference standard (Product # 20/136, available through The National Institute for Biological Standards and Control; www.nibsc.org) for semi-quantification. The assay was performed according to the manufacturer’s instructions. Test sample results were read off a standard curve returning relative units (RU) per mL. Values of > 8 RU/mL were considered positive.

### Receptor binding domain ELISA

The assay uses the SARS-CoV‑2 receptor binding domain (RBD) of the S1 subunit of the spike protein as target and an anti-human pan-Ig detector antibody (RBD pan-Ig; Wantai Biological, Bejing, China) for detection.

The procedure was done according to the manufacturer’s instructions with a slight modification by diluting previously determined positive samples 1:5 in phosphate buffered saline (PBS) in order to avoid a ceiling effect that occurred in the majority of undiluted samples. Assay read-outs (total Ig) are optical densities (OD) and results are reported as index values which were obtained by the ratio between the test sample OD and a reference sample OD provided with the test kit. Values of > 1 were considered positive.

### Nucleocapsid IgG ELISA

This assay targets the nucleocapsid protein (NCP) of SARS-CoV‑2 and was performed according to the manufacturer’s instructions (Euroimmun, Lübeck, Germany, Order # EI 2606-9601–2 G). Assay read-outs are anti-IgG optical densities (OD) and results are reported as index values which were obtained by the ratio between the test sample OD and the calibrator OD provided with the test kit. Index values of > 1.1 were reported positive.

### Anti-SARS-CoV-2 neutralizing antibody assays

Titers of neutralizing antibodies against SARS-CoV‑2 Wuhan strain were determined using vesicular stomatitis virus (VSV)-based assay and titers against SARS-CoV‑2 variants using a focus forming assay with replication competent SARS-CoV‑2 as previously described [11]. Briefly, for the VSV pseudovirus assay 4‑fold dilutions of heat-inactivated serum samples were incubated with a replication defective VSV expressing green fluorescent protein (GFP) as marker gene and pseudotyped with a C-terminally truncated version of the spike protein of SARS-CoV‑2 (Wuhan isolate) for 1 h. Consequently, 293T stably overexpressing human angiotensine converting enzyme (ACE)2 receptor, were infected with pseudovirus. For focus forming assay, replication competent SARS-CoV‑2 isolates, B.1.1.7 (alpha) variant (isolate C63.1, Innsbruck) or B.1.351 (beta) variant (isolate C24.1, Innsbruck) were pre-incubated with 4‑fold dilutions of heat-inactivated serum samples and mixes were subsequently used to infect Vero cells stably overexpressing transmembrane protease serine subtype 2 (TMPRSS2) and ACE2. Infected cells were counted using ImmunoSpot S6 Ultra‑V reader and CTL analyzer software (CTL Europe GmbH, Bonn, Germany). The 50% neutralization titers were calculated as highest plasma dilution where mean infection of duplicates lower than 50% of the mean of control wells with only virus. Samples with neutralization titers ≥ 1:16 were considered positive.

### Statistics

Analyses were done using GraphPad Prism software version 6.07 (GraphPad Software, La Jolla, CA USA,).

As most data were non-normally distributed, we report medians, interquartile and minimum to maximum ranges for descriptive analyses. Non-parametric analytical tests were used, i.e. Kruskal-Wallis test for repeated measures including Dunn’s multiple comparisons test and Spearman correlation coefficients.

The tolerated type 1 error was set at 5%.

### Ethics

The study was approved by the ethics committee of the Medical University of Innsbruck (https://www.i-med.ac.at/ethikkommission/) and all participants gave written informed consent.

## Results

### Epidemiology

No SARS-CoV‑2 re-infection occurred in any participant although there were 2 incidents of high-risk contacts. In one case the partner acquired COVID-19 and in the other case there was a close contact during a business meeting with an infected person not recognized at that time because of mild symptoms.

Between 10 and 12 months after SARS-CoV‑2 infection 3 participants received a single dose and 1 participant 2 doses of Comirnaty® (Pfizer, New York City, NY, USA; BNT162b2), and one person received a single dose of Vaxzevria® (AstraZeneca, Cambridge, UK; AZD1222).

### SARS-COV-2 antibody follow-up (primary predefined endpoint)

In the prospective cohort 29 individuals were followed up of whom 24 had blood collected at T1 (mean of 7 ± 2 weeks), and all 29 participants at T2 (mean of 14 ± 2 weeks) and T3 (mean of 27 ± 1 weeks). At T4 (mean of 50 ± 2 weeks) 2 participants were lost to follow-up because of being vaccinated meantime and not having had a sample withdrawn later than T3.

The number and relative proportion of antibody positive samples per assay and time point are summarized in Table 1.

**Table 1:** Number of positive samples (%) by follow-up time

Assay	T1	T2	T3	T4
NCP IgG	17/23 (74%)	17/28 (61%)	11/28 (39%)	5/26 (19%)
S1 IgG	23/24 (96%)	26/29 (90%)	23/29 (79%)	20/27 (74%)
RBD pan-Ig	23/24 (96%)	29/29 (100%)	29/29 (100%)	27/27 (100%)
NAb (wild type)	–	–	29/29 (100%)	25/27 (93%)
NAb (B.1.1.7)	–	–	–	13/26 (50%)
NAb (B.1.351)	–	–	–	6/26 (23%)

*T1* 1–2 months, *T2* 3–4 months, *T3* 6 months, *T4* 12 months after COVID-19

*NCP* nucleocapside, *IgG* Immunoglobulin G, *RBD* receptor binding domain, *NAb* neutralizing antibodies;

Binding antibodies against NCP showed the strongest decline over time followed by S1 with seropositivity rates of 19% and 74% at the 12-month follow-up, whereas antibodies targeting RBD remained stable until last follow-up.

Neutralizing antibodies against the SARS-CoV‑2 wild type persisted in the majority of participants with a conversion to negativity occurring in 2 cases at month 12. Wild type NAb titers did not change between T3 and T4 in roughly half of the participants and decreased by one titration step in the remainder. In contrast, at T4 NAb titers above threshold against variants B.1.1.7 and B1.351 occurred in 50% and 23% of unvaccinated participants.

**Fig. 1:**
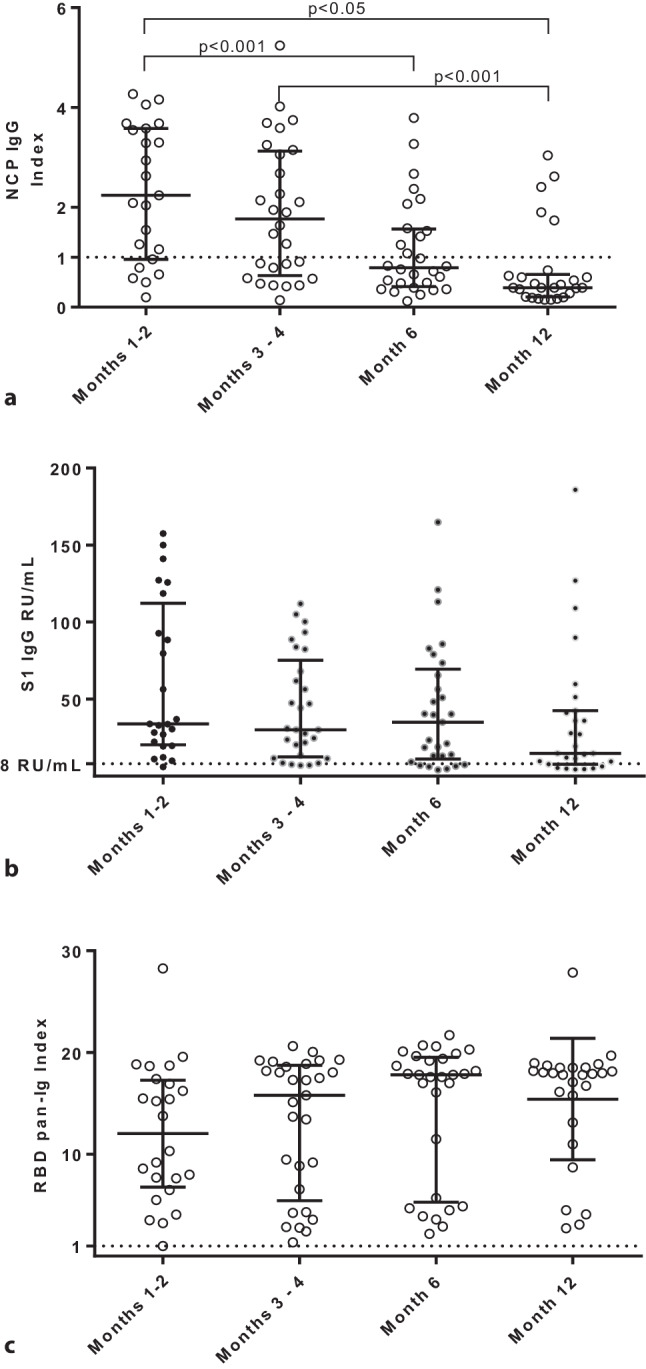
Anti-SARS-COV‑2 antibody values over time. Horizontal dotted lines indicate upper limits of negativity. **a** Antibody index values for the nucleocapsid (NCP) IgG assay at 4 consecutive time points. Lines indicate median values and error bars show interquartile ranges. Corrected *p*-values are shown above the brackets. **b** Antibody relative units/milliliter (RU/mL) values for the S1 IgG assay at 4 consecutive time points. Lines indicate median values and error bars show interquartile ranges. There were no significant differences across all time points. **c** Antibody index values for the receptor binding domain (RBD) pan-Ig assay at 4 consecutive time points. Lines indicate median values and error bars show interquartile ranges. There were no significant differences across all time points. Of note, at T1 (months 1–2) data of 4 individuals are missing

Median antibody values per time of follow-up are shown in Table 2 and their distribution is visualized in Fig. 1.

**Table 2:** Median antibody values by follow-up time (min-max range)

Assay	T1	T2	T3	T4	Post-vaccine
NCP IgG	2.24 (0.20–4.27)	1.77 (0.14–5.24)	0.79 (0.12–3.79)	0.39 (0.15–3.04)	–
S1 IgG	33.9 (6.0–157.5)	30.1 (6.9–111.9)	35.0 (4.0–164.8)	14.8 (4.3–185.9)	220 (210–253)
RBD pan-Ig	12.1 (1.0–28.3)	15.8 (1.4–20.6)	17.8 (2.2–21.7)	17.9 (2.7–27.9)	18.9 (18.2–19.4)
NAb (wild type) ^a^	–	–	1:64 (1:16–1:256)	1:16 (0–1:64)	1:1024 (1:256–1:1024)
NAb (B.1.1.7) ^b^	–	–	–	1:8 (0–1:64)	1:256 (1:256–1:1024)
NAb (B.1.351) ^b^	–	–	–	0 (0–1:64)	1:256 (1:256–1:1024)

*T1* 1–2 months, *T2* 3–4 months, *T3* 6 months, *T4* 12 months after COVID-19

^a^ performed by pseudovirus assay

^b^ performed by live virus assay

Significant differences occurred for NCP IgG between T1 vs. T3 and T4, and T2 vs. T4 (Dunn’s multiple comparisons) as well as between NAb titres between T3 and T4 (Wilcoxon test)

Post-vaccine titres were obtained from 5 participants

Antibody values over time by sex is shown in Figure S1. Overall, men had higher values early on with relatively equal values between sexes at last follow-up.

### Vaccine-induced antibody levels (secondary post hoc endpoint)

In 5 participants pre-vaccine and post-vaccine binding and neutralizing titres were measured. All NAb titres increased at least 16-fold (i.e. 2 titration steps) with minimum titres post-vaccination of 1:256. Results for wild type and variants are shown in Table 2.

### Correlations

There were strong and significant positive correlations within each binding antibody type between all time points ranging from r = 0.42 to r = 0.95. The closer to the last follow-up, the stronger the correlations with the month 12 values tended to be, except for NCP IgG indices. Also, there were strong and significant correlations between NAb titres (wild type) and S1 IgG RU/mL (r = 0.67, 95% CI: 0.48–0.80) as well as RBD pan-Ig indices (0.72, 95% CI: 0.55–0.83). NCP IgG antibodies correlated weakly and non-significantly with wild type NAb titres (r = 0.26, 95% CI: −0.01–0.50). Correlation cross-tabulations are shown in supplement table S1.

For the alpha variant NAb titres, there were significant correlations with S1 IgG RU/mL (r = 0.50, 95% CI: 0.13–0.75) as well as RBD pan-Ig indices (0.67, 95% CI: 0.38–0.84). Again, the correlation with NCP IgG values was non-significant and weak (0.18, 95% CI: −0.23–0.54).

We did not calculate correlations between beta variant NAb titres and binding antibodies because there were too few NAb positives.

## Discussion

Depending on the antigen we found a persistent anti-SARS-COV‑2 antibody response over 12 months after infection with a preference for the RBD and to a lesser degree for the entire S1 subunit of the spike protein. The S1 IgG assay seems somewhat insensitive, as of 7 samples that were formally negative at month 12 only 1 was also negative in the wild type neutralizing assay. The least sensitive antigen was the NCP which turned negative in most patients at month 12 and therefore has only limited use to distinguish between post-infectious and currently available vaccine-induced immunity, the latter exclusively targeting the spike protein. The NAb against SARS-CoV‑2 wild type, which was the predominant type during the first wave when the study participants contracted COVID-19, decreased at month 12 but was still detectable in 92% of convalescents. In contrast, NAb against the variants B.1.1.7 (the predominant variant in Austria at the time of last follow-up, https://www.ages.at/themen/krankheitserreger/coronavirus/sars-cov-2-varianten-in-oesterreich/) and B.1.351 were less frequently detected at last follow-up. The lower neutralizing capacity against variants is in line with previous observations [12]. The most common spike mutations of the current variants of concern are shown in table S2 which are probably responsible for partial immune evasion; however, there is a wide interindividual variation depending on the individual clonal composition of epitope-specific antibodies [10, 12].

The strong increase of binding and neutralizing antibodies including variants in convalescents after a single dose of vaccine has been described earlier with titres higher than in COVID-19 naïve persons after 2 doses of vaccine [13, 14]. Even post-vaccine, NAb titres against variants were slightly lower compared to the wild type with the limitation of a small sample size in our study; however, this is not consistently reported in the literature with some authors finding significantly higher NAb titres against the alpha variant than against the beta variant [5]. This is an important issue because NAb assays are variably standardized and therefore hardly comparable across different studies. Also, HLA polymorphisms determine the host’s immune response and variations of major histocompatibility complex (MHC) binding affinities depending on SARS-CoV‑2 variants and several HLA alleles have been shown [13]. There are quite a few other factors that need to be considered, such as age of subjects, sex, disease severity, and in cases of vaccine-induced immunization the interval between disease and vaccination or between vaccine doses, all of which are associated with the quantity, the quality, and the duration of the antibody response [4, 13–16]; however, all these observations underline the importance of the booster vaccinations in previously infected persons by broadening and increasing the immune response probably leading to long-term immunity.

The kinetics of binding antibodies are largely confirmative of previous findings with the most rapid and extensive decline occurring in the NCP IgG assay targeting the least immunogenic antigen as demonstrated by the weak correlation with NAb titres. Even early after disease only 74% of patients had levels above threshold for positivity, possibly indicating a sensitivity issue of this particular test system. This must be considered when using this assay for confirmation of possible infection after vaccination as such a strong decay did not occur using other systems [11].

For S1 IgG the decline of RU/mL values was less pronounced with 79% of patients remaining formally positive at the month 12 follow-up; however, the majority (6 of 7) of S1 IgG negative subjects had a positive result in the wild type NAb assay but only one was NAb positive against the alpha variant and none against the beta variant. On the other hand, of the S1 IgG positives at T4 roughly two thirds had a positive NAb titre against the alpha variant, and roughly one third against the beta variant corroborated by the strong correlation between at least the alpha variant and S1 IgG values.

In contrast to the above antigens, antibodies against RBD showed a stable development over 12 months after infection and correlated strongest with NAb titres. The fact that this assay not only determines IgG but also IgA and IgM might explain this observation apart from RBD seeming to be the strongest driver of humoral immunity [17]. Although IgG is the most long-lasting subclass some people also develop persistent IgM and IgA [10]. Clonal expansion of RBD-specific B‑cells and antibodies has been demonstrated and might also account for the quantitative increase of these antibodies as observed here [10]; however, one must be cautious because IgA and IgM assays might cross-react with non-SARS-CoV‑2 viral and bacterial agents causing respiratory infections [18].

Immunity as a clinical outcome has been described in early recordings of older dynasties of Egypt. It was, however, well-described by the historian Thucydides in his account of the Athenian plague of 430 B.C. [19]: “Yet it was with those who had recovered from the disease that the sick and dying found most compassion. These knew what it was from experience and had now no fear for themselves; for the same man was never attacked twice—never at least fatally.” The NAb tests as surrogates have been reported for COVID-19 finding a strong correlation between NAb titres and clinical vaccine response across seven trials [2], which needs to be interpreted with caution as there were different study designs and equivocal assay systems compared. Moreover, NAb assays only assess limited aspects of clinical immunity in that the interaction between test sera, viruses, and cultured cells susceptible to viral infection are included. There are several details that are not analyzed, such as cellular immunity overall, tissue-based cellular immunity [20], tissue-based humoral immunity with mucosal IgA not correlating well with circulating IgA [21] and of course immune memory [1, 22], which seems to be persisting as of now; however, using antibody tests as surrogates even if not fully validated seems more practical. Assessing long-term clinical immunity required probably 10,000s of unvaccinated convalescents followed prospectively over many years which seems hardly doable at least for the time being. Antibody concentrations at any time point correlated well with future antibody levels independent of the underlying assay. Although correlations are not formally predictive tools one can still assume that antibody persistency depends on the individual quantitative response. In more appropriate regression models others determined the half-life of protection based on NAb decay of 108 days [2]. One would probably rely on NAb titres to determine timing of booster vaccinations until more is known about the duration of clinical protection. In this context, international consensus should be sought, particularly on boosters of previously infected persons as there is broad immune response resulting in an even stronger response after one dose of vaccine [1, 13, 14, 22].

Sex differences with respect to antibody responses have been reported in different contexts [23–25] with somewhat conflicting results. Often, differential kinetics of titres were not considered. We found higher binding antibody levels in males shortly after infection with a stronger decrease for NCP IgG in females over time possibly related to the higher proportion more severely affected among males. For S1 IgG there was a similar but less pronounced pattern and for RBD pan-Ig antibodies females “caught up” during observation period, starting with lower antibody concentrations shortly after infection and finally slightly higher levels than males. Apparently, the more immune dominant antigens are driving a longer lasting immunity in females as described for influenza [23] and other anti-viral responses, only transiently however [26]. How this evolves long-term for SARS-CoV‑2 remains to be seen.

Finally, although one of the few prospective studies with repetitive testing over 12 months the limitation mainly confers to the relatively low number of participants, which is why we did not perform analytical statistics for some analyses, such as sex differences. Our findings are restricted to a previously healthy population with mild to moderate course of COVID-19 and to the age range between 24 and 64 years. The study was not designed to assess re-infection rates even though none occurred and protective immunity after COVID-19 has been demonstrated in larger populations [2]; however, there seems to be a longer lasting immunity after SARS-CoV‑2 infection than suggested by earlier reports [6] corroborated by a number of more recent publications [1, 4].

## Supplementary Information

Additional figures and tables.
