# Supplementary material for: 12-month SARS-CoV-2 antibody persistency in a Tyrolean COVID-19 cohort
Source: Wien Klin Wochenschr. 2021 Nov 23;133(23-24):1265–71. doi: 10.1007/s00508-021-01985-x (PMC8609251; doi:10.1007/s00508-021-01985-x)
Supplement: Supplementary file 1 — Additional figures and tables. [file 508_2021_1985_MOESM1_ESM.docx]

Supplement to Twelve-month SARS-CoV-2 antibody persistency in a Tyrolian Covid-19 cohort

**Figure 1S**

Panel A: Boxblots of NCP IgG indices by sex and time points after infection. Red boxes indicate females and blue boxes male patients. Lines within boxes indicate median values and borders range from 25 to 75 percentiles. Whiskers span from minimums to maximums. T1: 1-2 months, T2: 3-4 months, T3: 6 months, T4: 12 months after SARS-CoV-2 infection.

Panel B: Boxblots of S1 IgG RU/mL by sex and time points after infection. Red boxes indicate females and blue boxes male patients. Lines within boxes indicate median values and borders range from 25 to 75 percentiles. Whiskers span from minimums to maximums. T1: 1-2 months, T2: 3-4 months, T3: 6 months, T4: 12 months after SARS-CoV-2 infection.

Panel C: Boxblots of RBD pan-iG indices by sex and time points after infection. Red boxes indicate females and blue boxes male patients. Lines within boxes indicate median values and borders range from 25 to 75 percentiles. Whiskers span from minimums to maximums. T1: 1-2 months, T2: 3-4 months, T3: 6 months, T4: 12 months after SARS-CoV-2 infection.

Male versus female antibody responses

The longitudinal development of binding antibody titres differed between sexes as shown in the supplemental figure. NCP IgG antibody indices decreased stronger in female patients compared to males. Of 5 positive samples at T4 one (20%) was collected from a female participant. S1 IgG RU/mL decreased stronger in males with 5 of 15 samples (33%) reverting to negativity at T4 compared to 2 out of 14 (14%) females. In contrast, RBD pan-Ig indices were lower in female patients at T1 compared to males and increased over time whereas indices remained rather stable in males and were finally comparable at T4 in both sexes.

Of 13 female patients in whom NAb were tested at T4 12 (92%) had NAb against wild type, 9 (69%) against the alpha variant and 4 (30%) against the beta variant. Of 13 male patients 12 (92%) were NAb positive against wild type, 4 (31%) against the alpha variant, and 2 (15%) against the beta variant. This difference was independent of disease severity. Mild disease occurred in 12 (86%) and moderate disease in 2 (14%) of the female patients whereas mild disease course was seen in 7 (47%) and moderate disease in 8 (53%) male patients.

**Table S1: correlations**

Correlation coefficients for NCP IgG indices by time points

|  | Months 3 - 4 | Month 6 | Month 12 |
| --- | --- | --- | --- |
| Months 1-2 | 0,813 | 0,693 | 0,645 |
| Months 3 - 4 |  | 0,688 | 0,779 |
| Month 6 |  |  | 0,422 |

Correlation coefficients for S1 IgG RU/mL by time points

|  | Months 3 - 4 | Month 6 | Month 12 |
| --- | --- | --- | --- |
| Months 1-2 | 0,849 | 0,659 | 0,708 |
| Months 3 - 4 |  | 0,846 | 0,818 |
| Month 6 |  |  | 0,953 |

Correlation coefficients for RBD pan Ig indices by time points

|  | Months 3 - 4 | Month 6 | Month 12 |
| --- | --- | --- | --- |
| Months 1-2 | 0,734 | 0,603 | 0,529 |
| Months 3 - 4 |  | 0,854 | 0,602 |
| Month 6 |  |  | 0,619 |

**Table S2:** Overlapping Spike SNP across SARS-CoV-2 variants of concern

| Variant | SNP | | | | | |
| --- | --- | --- | --- | --- | --- | --- |
|  | L18F | K417N/T | E484K | N501Y | D614G | P681H/R |
| Alpha |  |  |  | x | x | x |
| Beta | x | x | x | x | x |  |
| Gamma | x | x | x | x | x |  |
| Delta |  | x |  |  | x | x |

Current variants of concern with overlapping spike mutations only. Mutations that occur in only one of the variants are not shown. Mutations in the RBD region are highlighted in light blue. Data obtained from Centers for Disease Control and Prevention (<https://www.cdc.gov/coronavirus/2019-ncov/variants/variant-info.html>), accessed on 28 September 2021.
